# RAB38 Facilitates Energy Metabolism and Counteracts Cell Death in Glioblastoma Cells

**DOI:** 10.3390/cells10071643

**Published:** 2021-06-30

**Authors:** Elena Bianchetti, Sierra J. Bates, Trang T. T. Nguyen, Markus D. Siegelin, Kevin A. Roth

**Affiliations:** Department of Pathology and Cell Biology, Columbia University Vagelos College of Physicians and Surgeons, New York, NY 10032, USA; bates.sierra@gmail.com (S.J.B.); tn2387@cumc.columbia.edu (T.T.T.N.); ms4169@cumc.columbia.edu (M.D.S.); kar2208@cumc.columbia.edu (K.A.R.)

**Keywords:** glioblastoma, RAB38, Bcl-xL, BH3-mimetics, statins

## Abstract

Glioblastoma is a high-grade glial neoplasm with a patient survival of 12–18 months. Therefore, the identification of novel therapeutic targets is an urgent need. RAB38 is a GTPase protein implicated in regulating cell proliferation and survival in tumors. The role of RAB38 in glioblastoma is relatively unexplored. Here, we test the hypothesis that RAB38 regulates glioblastoma growth using human glioblastoma cell lines. We found that genetic interference of RAB38 resulted in a decrease in glioblastoma growth through inhibition of proliferation and cell death induction. Transcriptome analysis showed that RAB38 silencing leads to changes in genes related to mitochondrial metabolism and intrinsic apoptosis (e.g., Bcl-xL). Consistently, rescue experiments demonstrated that loss of RAB38 causes a reduction in glioblastoma viability through downregulation of Bcl-xL. Moreover, RAB38 knockdown inhibited both glycolysis and oxidative phosphorylation. Interference with RAB38 enhanced cell death induced by BH3-mimetics. RAB38 antagonists are under development, but not yet clinically available. We found that FDA-approved statins caused a rapid reduction in RAB38 protein levels, increased cell death, and phenocopied some of the molecular changes elicited by loss of RAB38. In summary, our findings suggest that RAB38 is a potential therapeutic target for glioblastoma treatment.

## 1. Introduction

Glioblastomas cause the death of roughly 13,000 Americans per year [1]. The majority of glioblastoma patients die within one year of diagnosis and only 4.7% live beyond two years [2,3,4]. Current glioblastoma treatments are largely limited to surgical resection followed by chemotherapy and radiation [5]. While numerous clinical trials are underway to test novel treatment regimens, results to date have been disappointing. Therefore, there is a critical need to identify novel therapeutic targets in an effort to develop new treatment regimens with better outcomes.

The RAB family of small GTPases is part of the Ras protein superfamily [6,7] and is primarily responsible for regulating intracellular vesicle transport and migration. The RAB32 subfamily contains two proteins, RAB32 and RAB38, and is known to regulate endosome-mediated membrane trafficking of lysosome-related organelles [6]. RAB32 and RAB38 are genetically similar and structural analogs; however, RAB32 is noted to be more broadly expressed than RAB38 [6]. RAB38, also known as rrGTPbp and NY-MEL-1 [8], has been reported to be upregulated in glioblastoma, and high levels of its expression are associated with a poor prognosis and enhanced cell migration [9]. When comparing low-grade gliomas to glioblastomas, the latter demonstrated significantly higher RAB38 expression [9]. Furthermore, high expression of RAB38 in low-grade gliomas is correlated with a poor prognosis [9].

Despite the evidence that RAB38 is upregulated in gliomas, the functional significance of RAB38 in glioblastoma growth and protection from cell death has not been elucidated. Here, we examine the effects of loss of RAB38 function in human glioblastoma cell cultures. We test the overarching hypothesis that RAB38 plays an important prosurvival role in glioblastoma cells. We found that knockdown of RAB38 results in increased cell death and loss of mitochondrial membrane potential coupled with decreased oxygen consumption rate and glycolytic activity. Based on the inhibitory effects on mitochondrial metabolism, we tested BH3-mimetics in the setting of RAB38 silencing and made the novel discovery that dual inhibition of Bcl-2/Bcl-xL and RAB38 causes synergistic reduction in cellular viability. Moreover, we found that FDA-approved statins lead to a rapid reduction in RAB38 levels and phenocopy the effects of RAB38 loss of function. These results position RAB38 as a potential novel therapeutic target for gliomas.

## 2. Materials and Methods

### 2.1. Prognostic Significance of RAB38 Expression Levels

The present study used OncoLnc (www.oncolnc.org/, accessed on 25 November 2020) online tools to assess the impact of RAB38 mRNA levels on patient survival in glioblastoma and low-grade gliomas (LGG).

### 2.2. Reagents

For Western blotting, we used primary antibodies for RAB38 (Cell Signaling Technology, Danvers, MA, USA; #14365), RAB32 (Sigma, St. Louis, MO, USA; #010m4795), OXPHOS (Abcam, Cambridge, MA, USA; #ab110411), C-Myc (Cell Signaling Technology, Danvers, MA, USA; #5605), Mcl-1 (Cell Signaling Technology, Danvers, MA, USA; #5453S), Bcl-2 (Cell Signaling Technology, Danvers, MA, USA; #4223), Bcl-xL (Cell Signaling Technology, Danvers, MA, USA; #2764S), and β-actin (Sigma, St. Louis, MO, USA; #076M4786V) and secondary antibodies for HRP-conjugated goat anti-mouse (Thermo Scientific, Waltham, MA, USA; #31430) and goat anti-rabbit (Thermo Scientific, Waltham, MA, USA; #31460). For cell death assays, the positive control staurosporine was obtained from Sigma-Aldrich (St. Louis, MO, USA; STS #S5921). For drug treatments, simvastatin was purchased from Selleckchem (Houston, TX, USA; #S1792), lovastatin from MedChemExpress (South Brunswick, NJ, USA; #HY-N0504), and ABT263 from Selleckchem (Houston, TX, USA; #S1001).

### 2.3. Cell Culture

We used human astrocytes (ScienceCell Research Laboratories, Carlsbad, CA, USA; #1800), human glioblastoma cell lines LN229 and T98G (ATCC, Manassas, VA, USA), and the GBM43 patient-derived xenograft (PDX) cell line (Mayo Clinic Brain Tumor Patient-Derived Xenograft National Resource). Cells were maintained in Dulbecco’s modified Eagle’s medium with primocin (DMEM; Corning, Manassas, VA, USA), containing 1% primocin (Invivogen, San Diego, CA, USA) and 10% fetal bovine serum unless otherwise noted (FBS; Hyclone, Logan, UT, USA). Cells were kept in an incubator (37 °C in a humidified 5% CO_2_, 95% air environment) on uncoated Petri dishes and monitored regularly for density and doubling time. Astrocytes were maintained using the same medium with the addition of N_2_ (Thermo Fisher Scientific, Waltham, MA, USA; #2135).

### 2.4. RNAi 

Twenty-four hours after plating, cells were transfected as previously described [10]. Transfection was performed according to the provided kit protocol including dose-dependency without fetal bovine serum and without antibiotics (Dharmacon ON-TARGETplus Non-targeting Pool, #D-001810-10-05, Human RAB38, #L-010059-00-0010). Twenty-four hours after transfection, fetal bovine serum was added for a total concentration of 10%. Successful transfection was confirmed after 24, 48, and 72 h using Western blotting analysis. 

### 2.5. Cell Viability Assays

ATP levels present were recorded using CellTiter-Glo assay according to the recommended protocol by the manufacturer (Promega, Madison, WI, USA; #G7571). A 96-well plate was seeded with 2000 cells per well, and transfection was performed at 24, 48, and 72 h prior to analysis. In addition, a 96-well plate was seeded with 250 cells per well, transfection was performed 24 h after plating, and the viability analysis was performed after 14 days. Following transfection, 100 µL of CellTiter-Glo assay reagent was added to each well along with 100 µL of complete medium. Cells were incubated at room temperature for 10 min prior to analysis through a plate reader (SpectraMax i3x multi-mode detection platform, Molecular Devices, San Jose, CA, USA).

### 2.6. Western Blotting

Cells were seeded on 12-well plates in a number of 30,000/well. Samples for the Western blots were collected using Laemmli buffer containing 5% 2-mercaptoethanol (Bio-Rad, Hercules, CA, USA; #161-0710) and 95% 2x Laemmli Sample Buffer (Bio-Rad, Hercules, CA, USA; #161-0737), and additionally, a protease inhibitor cocktail was added (Thermo Scientific, Waltham, MA, USA; #1861281). The medium was removed and cells were lysed directly in the buffer following washing with PBS. RAB38 and RAB32 primary antibodies were diluted 1:100, β-actin was diluted 1:8000, and all other primary antibodies were diluted 1:500. Secondary antibodies were diluted 1:5000. Pierce ECL Western blotting substrate was used for detection (chemiluminescence) (Pierce ECL; Thermo Scientific, Waltham, MA, USA). The membranes were developed with an Azure c-300 Gel Doc and Western blot imaging (Azure Biosystem, Dublin, CA, USA).

### 2.7. Measurements of Cell Proliferation

To determine the effect of RAB38 silencing on the cell cycle, 10,000 cells were seeded and, the day after, transfected with nontargeting (siNT) or RAB38-specific siRNA (siRAB38) for 72 h. Cells were detached with trypsin and fixed in cold 85% ethanol for 4 h and then stained with propidium iodide (PI) solution (PI/RNAse Staining Solution, 4087, Cell Signaling, Danvers, MA, USA) at room temperature for 15 min. Fluorescence was measured using a BD FACSCalibur flow cytometer. The percentage of cells in each phase of the cell cycle was determined using FlowJo software (version 8.7.1; Tree Star, Ashland, OR, USA).

### 2.8. Measurements of Apoptosis and Mitochondrial Stress

Cells were seeded in each well of a 12-well plate in a number of 10,000/well and transfected, then permitted to grow for 48 and 72 h. Annexin V/propidium iodide staining was performed according to the recommended protocol of the FITC Annexin V Apoptosis Detection Kit I (BD Pharmingen, San Diego, CA, USA). Mitochondrial membrane potential was measured via a tetramethylrhodamine ethyl ester perchlorate (TMRE) staining performed according to the recommended protocol of the CellSimple Mitochondrial Membrane Potential Assay Kit II (Cell Signaling, Danvers, MA, USA; #45898). A BD FACSCalibur flow cytometer was used to obtain fluorescence data, and subsequent analysis was performed with the FlowJo software.

### 2.9. Measurements of Cell Metabolism

Oxygen consumption rates (OCR) and glycolytic activity (ECAR) were measured using Seahorse Agilent Technologies XF Cell Mito Stress Test Kit and following the provided protocol (Agilent Technologies, Cedar Creek, TX, USA; #103015-100 and #103017-100). 10,000 cells/well were seeded in a 12-well plate and transfected 24 h after plating using nontargeting or RAB38 siRNA. The medium was supplemented with 1.5% FBS the day after, and cells were moved from the 12-well plate to the 8-well cell culture miniplate. Analyses were performed 48 h after transfection using a Seahorse Bioscience extracellular flux analyzer.

### 2.10. Transcriptome Analysis

Twenty-four hours after transfection, fetal bovine serum was added for a total concentration of 1.5%. Forty-eight hours after transfection, RNA was extracted using RNeasy Mini Kit (Qiagen, Hilden, Germany; Cat. No. 74104). Microarray and subsequent gene set enrichment analysis were performed as described earlier [11]. The data discussed in this publication have been deposited in NCBI’s Gene Expression Omnibus and are accessible through GEO Series accession number GSE162444 (https://www.ncbi.nlm.nih.gov/geo/query/acc.cgi?acc=GSE162444, accessed on 21 June 2021).

### 2.11. Overexpression of Human c-Myc and Bcl-xL

Adenoviruses carrying human Bcl-xL (Ad-h-BCL2L1; #ADV-202038) and human c-Myc (Ad-c-Myc; #1285) and control virus (Ad-null; #1240) were purchased from Vector Biolabs (Philadelphia, PA, USA). LN229 cells were seeded at a density of 50,000 cells/well in a 12-well dish. The day after, the adenoviral infection was performed at a multiplicity of infection of 12.5 for c-Myc and 200 for Bcl-xL, using a volume of 500 µL of DMEM in each well. The virus solution was replaced with complete medium 19 h later. Subsequently, cells were plated at a density of 2000 cells/well in a 96-well plate for the viability assay and 10,000 cells/plate in a 12-well dish for flow cytometry analysis.

### 2.12. Statistics

All data are presented as mean ± SD unless otherwise indicated; *n* = 3 notation refers to three independent experiments. Statistical significance was determined using ANOVA and *t*-test analysis with a significance threshold of *p* = 0.05. * *p* < 0.05; ** *p* < 0.01; ***/**** *p* < 0.001, n.s. means not significant. GraphPad Prism 8 software was used for statistical analysis. Western blot image quantification and comparisons were performed using ImageJ. Bliss analysis was performed to detect synergistic, additive, or antagonistic effects as described previously [12].

## 3. Results

### 3.1. RAB38 Is a Prognostic Marker in Glioblastoma and Genetic Interference with RAB38 Affects Cellular Viability of Glioblastoma Cultures

**Figure 1:**
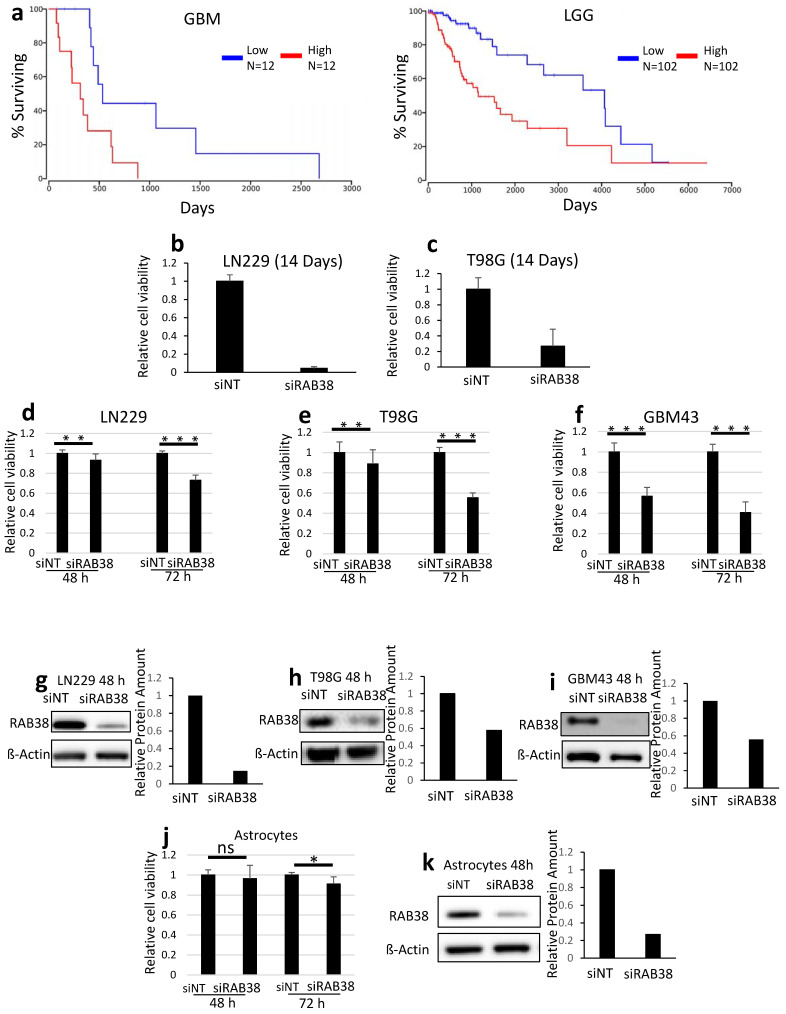
RAB38 silencing reduces the viability of glioblastoma cell lines. (**a**) Survival curves for high and low RAB38 expressing tumors in GBM and LGG (TCGA database) are shown. (**b**–**c**) LN229 (**e**) and T98G (**f**) were transfected with nontargeting (NT) or RAB38-specific siRNA (siRAB38). After 14 days, cellular viability was determined by CellTiter-Glo assay and relative cell viability was calculated. Data are presented as mean and SD, *n* = 3. (**d**–**f** and **j**) LN229 (**d**), T98G (**e**), GBM43 (**f**), and astrocytes (**j**) were transfected with nontargeting (NT) or RAB38-specific siRNA (siRAB38) for 48 and 72 h. Cellular viability was determined by CellTiter-Glo assay and relative cell viability was calculated. Data are presented as mean and SD, *n* = 3. (**g**–**I**,**k**) LN229 (**g**), T98G (**h**), and GBM43 cells (**i**) and astrocytes (**k**) were transfected with nontargeting (siNT) or RAB38-specific siRNA and whole-cell protein extracts were examined by Western blot analysis for RAB38 expression. β-Actin served as a loading control. Bar graphs display protein quantification levels determined by ImageJ (mean and SD presented). * *p* < 0.05; ** *p* < 0.01; ***/**** *p* < 0.001, n.s. means not significant.

We interrogated the TCGA database to assess the prognostic impact of high levels of RAB38 mRNA in glioblastoma patients and patients with low-grade gliomas. Cox regression results for RAB38 (http://www.oncolnc.org/, accessed on 25 November 2020) indicated that expression of RAB38 highly correlated with a decreased overall survival in both glioblastoma (*p*-value = 3.40 × 10^−2^) and LGG tissues (*p*-value = 2.40 × 10^−6^) (Figure 1a). In our studies, we found that human astrocytes and our panel of glioblastoma cultures demonstrated RAB38 protein expression (data not shown). To determine if RAB38 could play a potential role in regulating glioblastoma cell death and survival, we examined the effects of RAB38 silencing on the human glioblastoma established cell lines LN229 and T98G and on the patient-derived cell line GBM43. siRNA pool, made by four individual siRNAs, was used to silence RAB38 gene expression. Glioblastoma cell viability 14 days following transfection of RAB38 siRNA was significantly decreased compared to nontargeting siRNA controls (Figure 1b,c). Examination of cells at earlier time points showed that siRNA silencing of RAB38 produced a significant decrease in viability of LN229, T98G, and GBM43 cell lines 48 h following transfection, which becomes strongest at 72 h (Figure 1d–f). To confirm a successful knockdown of RAB38, Western blotting was performed and showed a marked reduction in RAB38 protein levels in LN229 (Figure 1g), T98G (Figure 1h), and GBM43 (Figure 1i) 48 h after transfection. To determine if RAB38 could be a selective therapeutic target for glioblastoma cells, we examined the effects of RAB38 silencing on astrocytes isolated from human cerebral cortex. Examination of the cells at 48 and 72 h after transfection showed that siRNA silencing of RAB38 modestly affects the viability of astrocytes only 72 h after silencing (Figure 1j). To confirm a successful knockdown of RAB38, a Western blot was performed and showed a consistent reduction in RAB38 protein levels 48 h after transfection (Figure 1k). The ANOVA combined with the multiple comparisons between groups (astrocytes vs. all others) shows a significant difference in cellular viability following RAB38 silencing in astrocytes versus the three GBM lines tested (Supplementary Figure S1a). 

### 3.2. RAB38 Knockdown Induces a Decrease in c-Myc Protein Levels Accompanied by Changes in Cell Cycle Progression

Transcriptome analysis followed by gene set enrichment analysis (GSEA) suggested that RAB38 impairment correlates with low levels of c-Myc targets in LN229 cells (Supplementary Figure S1b). Therefore, we focused our attention on c-Myc, a transcription factor that plays an important role in tumor proliferation [13] and cell cycle regulation by facilitating the transition from the G1 phase to the S phase [14,15]. Western blotting displayed a significant reduction in c-Myc protein levels 48 or 72 h after RAB38 inhibition in LN229 (Supplementary Figure S1c), T98G (Supplementary Figure S1d), and GBM43 (Supplementary Figure S1e) cells. Cell cycle analysis revealed that glioblastoma cells transfected with nontargeting siRNA exhibited regular cell cycle patterns (Supplementary Figure S1f,g). In contrast, LN229 and T98G cells transfected with RAB38 siRNA exhibited a greater percentage of cells in the G1 phase and fewer cells in the S phase, suggesting an inhibition of cell cycle progression (Supplementary Figure S1f,g). To explore if c-Myc has a role in the change in cell cycle progression and the associated inhibition of proliferation induced by RAB38 silencing, we overexpressed c-Myc in LN229 cells, using an adenovirus (Supplementary Figure S1h–j). Western blot analysis showed a dose-dependent increase in c-Myc protein levels at 6.25 and 12.5 MOI (multiplicity of infection; the number of virions that are added per cell during infection) compared to the control virus (Supplementary Figure S1h). Overexpression of c-Myc rescued from RAB38-silencing-mediated reduction in cellular viability in LN229 (Supplementary Figure S1i,j). Consistently, cell cycle analysis demonstrated that overexpression of c-Myc partially protected the cells from RAB38-silencing-mediated inhibition of cell cycle progression (Supplementary Figure S1k).

### 3.3. RAB38 Knockdown Causes Cell Death in Glioblastoma Cell Lines, but Not in Human Astrocytes

**Figure 2:**
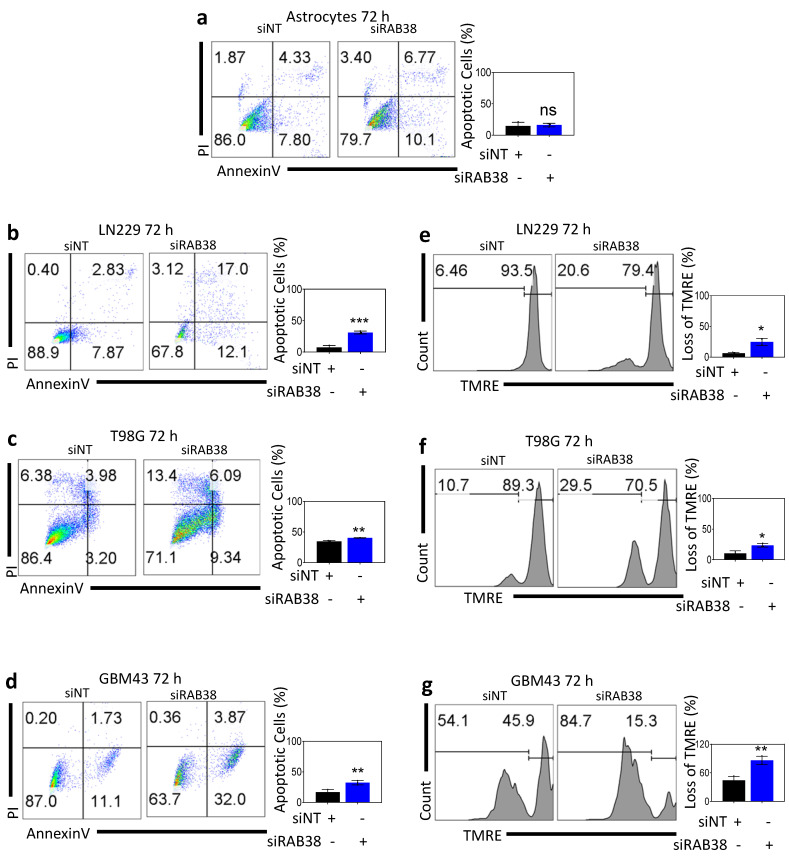
Loss of RAB38 function causes glioblastoma cell death associated with loss in mitochondrial membrane potential. (**a**–**d**) Astrocytes (**a**), LN229 (**b**), T98G (**c**), and GBM43 (**d**) glioblastoma cell lines were transfected with either NT or RAB38 siRNA and after 72 h were stained with annexin V/propidium iodide and analyzed by flow cytometry (representative plots shown). Representative plots and a histogram with statistical analysis are presented (*n* = 3). (**e**–**g**) LN229 (**e**), T98G (**f**), and GBM43 (**g**) cells were transfected with either siNT or siRAB38 and after 72 h were stained with TMRE and analyzed by flow cytometry (representative plots shown). Representative plots and a histogram with statistical analysis are presented (*n* = 3). * *p* < 0.05; ** *p* < 0.01; ***/**** *p* < 0.001, n.s. means not significant.

We performed an annexin V/PI assay to determine if silencing of RAB38 induced apoptotic cell death. In astrocytes, the nontargeting control group showed approximately 86% viable cells (annexin V low, PI low) with approximately 8% of cells in early apoptosis (annexin V high, PI low) and 4% of cells in late stages of cell death (annexin V high, PI high) at 72 h (Figure 2a). Likewise, cells exposed to RAB38 silencing exhibited approximately the same percentage of cells in early and late stages of cell death, respectively (Figure 2a). The LN229 nontargeting control group showed approximately 89% viable cells (annexin V low, PI low) with approximately 8% of cells in early apoptosis (annexin V high, PI low) and 3% of cells in late stages of cell death (annexin V high, PI high) at 72 h (Figure 2b). In contrast, LN229 cells exposed to RAB38 silencing exhibited approximately 68% viability with 12% and 17% of cells in early and late stages of cell death, respectively (Figure 2b). After RAB38 silencing, T98G and GBM43 cells showed increased cell death, with a peak of 16% of cells undergoing death 72 h after silencing for T98G (Figure 2c) and 36% for GBM43 (Figure 2d). To determine if there was an increase in ongoing mitochondrial damage after RAB38 knockdown, we performed a tetramethylrhodamine ethyl ester perchlorate (TMRE) assay. Control cells with nontargeting treatment showed few cells with loss of mitochondrial membrane potential. In contrast, following RAB38 knockdown, there was a time-dependent loss of mitochondrial membrane potential (Figure 2e–g).

### 3.4. RAB38 Knockdown Decreases Metabolic Activity

**Figure 3:**
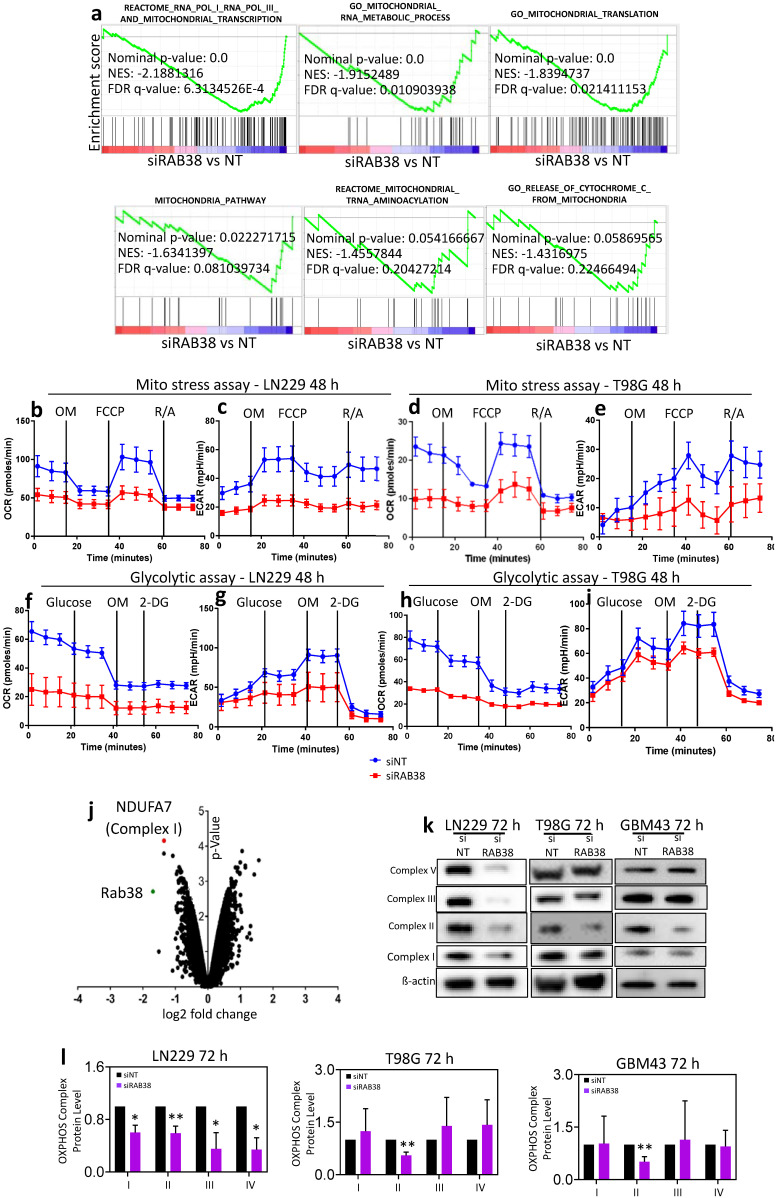
RAB38 silencing leads to inhibition of mitochondrial energy metabolism (**a**). LN229 cells were transfected with nontargeting or RAB38 siRNA. RNA was extracted 48 h after transfection. Microarray analysis with subsequent gene set enrichment analysis (GSEA) was performed. Shown are GSEA plots with downregulation of genes related to mitochondrial metabolism and the respective statistical analysis. *n* = 2. (**b**–**e**) LN229 (**b**–**c**) and T98G (**d**–**e**) cells were transfected with nontargeting or RAB38 siRNA. Forty-eight hours after transfection, the oxygen consumption (OCR) and extracellular acidification rate (ECAR) were measured on a seahorse analyzer (Seahorse XF Cell Mito Stress assay). Representative plots are shown, *n* = 3. (**f**–**i**) LN229 (**f**–**g**) and T98G (**h**–**i**) cells were transfected with nontargeting or RAB38 siRNA. Forty-eight hours after transfection, the oxygen consumption (OCR) and extracellular acidification rate (ECAR) were measured on a seahorse analyzer (Seahorse XF Cell Glycolytic Stress assay). Representative plots are shown, *n* = 3. (**j**) Volcano plot depicting significantly up- and downregulated genes following transfection with nontargeting or RAB38-specific siRNA for 48 h in LN229 cells. Highlighted are the mRNA levels of NDUFA7 (complex I, in red) and RAB38 (in green). FC: fold change. (**k**) RAB38 knockdown was performed in LN229, T98G, and GBM43, and whole-cell protein extracts were examined by Western blot analysis of RAB38 (data not shown) and OXPHOS complexes I, II, III, and V. β-Actin Western blot analysis was performed to confirm equal protein loading. (**l**) Protein quantification was analyzed through ImageJ. Representative plots and a histogram with statistical analysis are presented (*n* = 3). * *p* < 0.05; ** *p* < 0.01.

Based on our RNA transcriptome analysis and GSEA, groups of genes related to mitochondrial metabolism were suppressed or impaired by RAB38 silencing (Figure 3). For this reason and given that metabolic perturbations are linked with cellular proliferation and cell death, we interrogated mitochondrial energy metabolism in our model systems. We performed extracellular flux analysis to determine if there were changes in metabolic activity of glioblastoma cells following RAB38 knockdown. A mitochondrial stress assay showed a significantly decreased oxygen consumption rate in cells transfected with RAB38 siRNA compared to respective controls (Figure 3b,d). Extracellular acidification rate, a measure of glycolysis, was also significantly decreased (Figure 3c,e). To this end, we performed a glycolytic stress assay. Cells transfected with RAB38 siRNA were characterized by a significantly decreased extracellular acidification rate as compared to control transfected cells (Figure 3f,h). Akin to the mitochondrial stress assay, the oxygen consumption rate was also significantly decreased (Figure 3g,i). Taken together, these results show that RAB38 knockdown decreases metabolic activity through a reduction in both oxidative phosphorylation and glycolysis. In alignment with this metabolic phenotype, interrogation of our microarray data, involving silencing of RAB38 in LN229 cells, demonstrated significantly lower levels of NDUFA7 (a component of the OXPHOS complex I) (Figure 3j), suggesting a potential impairment of the electron transport chain in response to RAB38 suppression. These results were confirmed on the protein level as well. Following RAB38 silencing, LN229 cells showed decreased protein levels of OXPHOS complexes I, II, III, and V, compared to the nontargeting control transfected cells (Figure 3k,l). The downregulation of the complexes was more pronounced in the LN229 cell line as compared to the T98G and GBM43 lines (Figure 3k). In addition, we noted that RAB38 silencing appeared to affect the complex II protein levels most consistently across all three cell lines (Figure 3k,l).

### 3.5. RAB38 Knockdown Reduces the Protein Expression of Antiapoptotic Bcl-2 Family Members and Overexpression of Bcl-xL Rescues from Cell Death Induced by Loss of RAB38 Function

**Figure 4:**
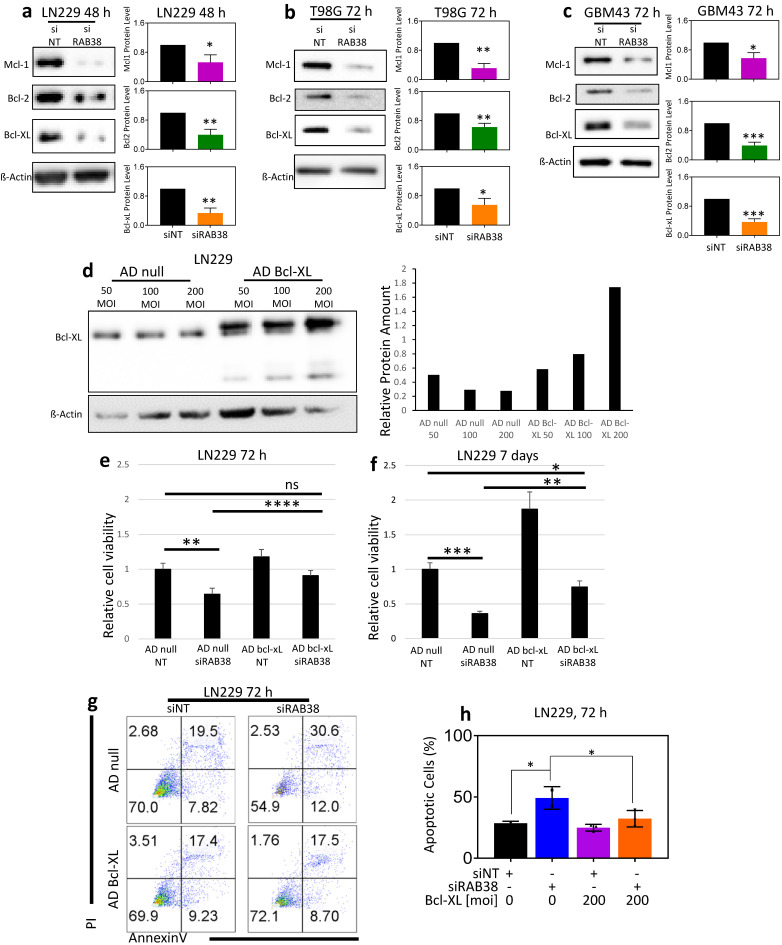
RAB38 silencing downregulates the protein expression of antiapoptotic Bcl-2 family members. (**a**–**c**) RAB38 knockdown was performed in LN229 (**a**), T98G (**b**), and GBM43 (**c**), and whole-cell protein extracts were examined by Western blot analysis of Mcl-1, Bcl-2, Bcl-xL, and RAB38. β-Actin Western blot analysis was performed to confirm equal protein loading. Protein quantification was analyzed through ImageJ. Representative plots and a histogram with statistical analysis are presented (*n* = 3). (**d**) Bcl-xL overexpression was performed in LN229 using an adenovirus, and whole-cell protein extracts were examined by Western blot analysis of Bcl-Xl and β-actin. Bar graphs display protein quantification levels determined by ImageJ. (**e**,**f**) LN229 cells were transfected with nontargeting (NT) or RAB38-specific siRNA (siRAB38) using either cells overexpressing Bcl-xL or controls. After 72 h (**e**) or 7 days (**f**), cellular viability was determined by CellTiter-Glo assay and relative cell viability was calculated. Data are presented as mean and SD, *n* = 3. (**g**,**h**) LN229 glioblastoma cells overexpressing Bcl-xL were transfected with either NT or RAB38 siRNA and stained with annexin V/propidium iodide at 72 h, then analyzed using flow cytometry. Representative plots and a histogram with statistical analysis are presented (*n* = 3). * *p* < 0.05; ** *p* < 0.01; ***/**** *p* < 0.001, n.s. means not significant.

Since the Bcl-2 family members are important regulators of cell death and our GSEA pointed towards dysregulation of mitochondrial function, we determined the protein levels of Bcl-2, Bcl-xL, and Mcl-1 after RAB38 silencing in all cell lines (Figure 4a–c). We found a suppression of all three proteins following RAB38 silencing in all cell lines (Figure 4a–c). Since Bcl-xL has been shown to be the key mediator of intrinsic apoptosis in solid malignancies, we focused our rescue studies on this protein. In order to explore if Bcl-xL has a role in the cell death induced by RAB38 silencing, we overexpressed Bcl-xL in the LN229 cell line, using an adenovirus (Figure 4d–h). To confirm a successful overexpression of Bcl-xL, a Western blot was performed and showed a marked and dose-dependent increase in Bcl-xL protein levels at 50, 100, and 200 MOI compared to controls (Figure 4d). Viability of LN229 cells overexpressing Bcl-xL at 72 h following RAB38 silencing was significantly increased compared to cells with normal levels of Bcl-xL (Figure 4e), and this result was maintained 7 days following transfection (Figure 4f). We performed both annexin V/PI and cellular viability assays to determine if Bcl-xL has a role in cell death induced by RAB38 silencing (Figure 4g–h). Indeed, our results show that Bcl-xL overexpression rescues both reduced cell proliferation and increased cell death elicited by RAB38 silencing (Figure 4e–h).

### 3.6. RAB38 Knockdown Sensitizes Glioblastoma Cell Lines for a BH3-Mimetic Drug

**Figure 5:**
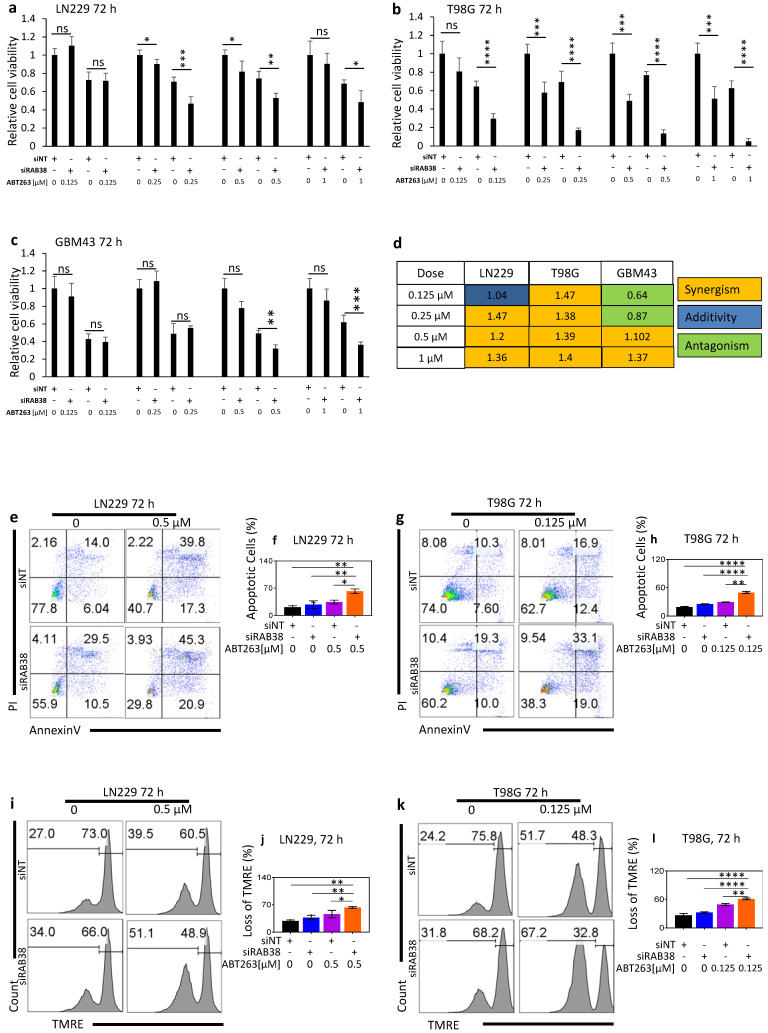
RAB38 knockdown renders glioblastoma cells vulnerable to a BH3-mimetic drug enhancing cell death in vitro. (**a**–**c**) LN229 (**a**), T98G (**b**), and GBM43 (**c**) cells were transfected with nontargeting (NT) siRNA or RAB38 siRNA. Thereafter, cells were incubated with vehicle or ABT263, for 72 h. ABT263 was used at four different concentrations. Cellular viability was determined by CellTiter-Glo assay and relative cell viability was calculated. Data are presented as mean and SD, *n* = 3. (**d**) Bliss analysis was performed in order to understand whether RAB38 silencing and ABT263 had a synergistic effect on the reduction in cellular viability. (**e**–**h**) LN229 (**e**) and T98G (**f**) glioblastoma cell lines were transfected with either siNT or RAB38 siRNA. Thereafter, transfected cells were treated with vehicle or ABT263, stained with annexin V/propidium iodide 72 h after silencing, and then analyzed by flow cytometry. Representative plots and a histogram with statistical analysis are presented (*n* = 3). (**i**–**l**) LN229 (**c**) and T98G (**d**) cells were transfected with either siNT or siRAB38 siRNA and treated with vehicle or ABT263. Seventy-two hours after transfection, cells were labeled with TMRE and analyzed by flow cytometry. Representative plots and a histogram with statistical analysis are presented (*n* = 3). * *p* < 0.05; ** *p* < 0.01; ***/**** *p* < 0.001, n.s. means not significant.

Prior studies from others and our own laboratory clearly demonstrate that silencing of Mcl-1 enhances the killing effect of the classical BH3-mimetic, ABT263 [11]. Since it has been shown by us and others that inhibition of mitochondrial metabolism primes tumor cells to apoptosis induction by BH3-mimetics [16], we tested whether or not interference with RAB38 enhances the cytotoxic effects of ABT263 (a Bcl-2/Bcl-xL inhibitor). Indeed, we found that RAB38 silencing enhanced the reduction in viability elicited by ABT263 (Figure 5a–c). We evaluated whether this occurred in a synergistic manner and noted that in most instances the interaction between Bcl-2/Bcl-xL and RAB38 inhibition resulted in synergistic growth reduction (Figure 5d). We conducted flow cytometric analyses to elucidate whether part of the mechanism responsible for the enhanced growth inhibition of a combined treatment with ABT263 and RAB38 silencing involved the induction of cell death with apoptotic features. Flow cytometry analysis led to the observation that LN229 cells with RAB38 silencing developed typical features of apoptosis, which was further enhanced in the presence of ABT263 treatment, showing a significantly increased fraction of annexin V-positive cells (apoptotic cells) (Figure 5e–h). Moreover, RAB38 knockdown in combination with ABT263 treatment resulted in enhanced loss of mitochondrial membrane potential (Figure 5i–l).

### 3.7. Statins Cause Glioblastoma Cell Death and Induce a Decrease in RAB38, as Well as c-Myc, Mcl-1, Bcl-2, and Bcl-xL, In Vitro

**Figure 6:**
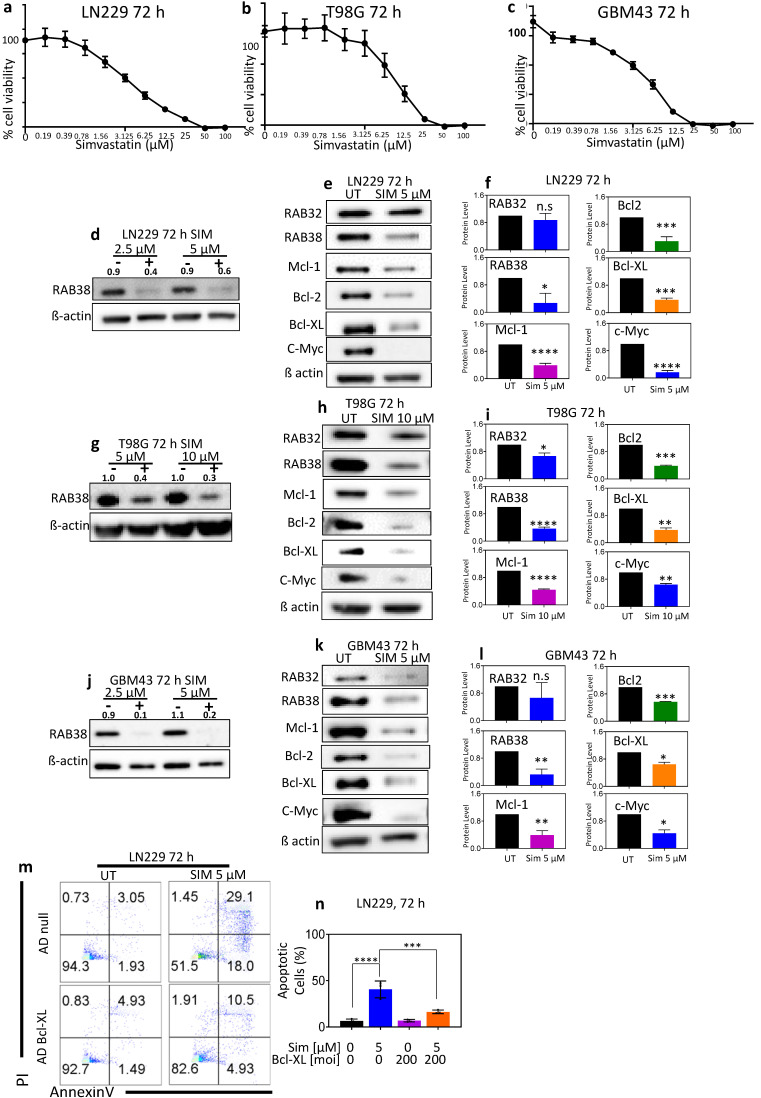
Simvastatin causes a reduction in cellular viability and downregulates RAB38, RAB32, Mcl-1, Bcl-2, Bcl-xL, and c-Myc protein expression. (**a**–**c**) LN229 (**a**), T98G (**b**), and GBM43 (**c**) glioblastoma cell lines were treated with increasing concentrations of simvastatin for 72 h. Cellular viability was determined by CellTiter-Glo assay and the IC50 values were calculated based on a nonlinear regression analysis. Data are presented as mean and SD, *n* = 3. (**d**–**l**) LN229 (**d**–**f**), T98G (**g**–**i**), and GBM43 (**j**–**l**) were treated with simvastatin at two different concentrations for 72 h. Whole-cell protein extracts were analyzed by Western blot for RAB38, RAB32, Mcl-1, Bcl-2, Bcl-xL, and c-Myc. β-Actin Western blot analysis was performed to confirm equal protein loading. Protein quantification was analyzed through ImageJ. Representative plots and a histogram with statistical analysis are presented (*n* = 3). (**m**–**n**) LN229 cells transduced with a control adenovirus or an adenovirus encoding Bcl-xL were treated with simvastatin, stained with annexin V/propidium iodide at 72 h following treatments, and analyzed by flow cytometry. Representative plots and a histogram with statistical analysis are presented (*n* = 3). * *p* < 0.05; ** *p* < 0.01; ***/**** *p* < 0.001, n.s. means not significant.

RAB family members, including RAB38, require post-translational prenylation for activity. To assess the potential efficacy of prenylation inhibition in treating glioblastoma in vitro, we tested the effects of simvastatin and lovastatin, two FDA-approved prenylation inhibitors. Our studies demonstrated that treatment of glioblastoma cells with simvastatin or lovastatin caused a decrease in cell viability in LN229, T98G, and GBM43 cells in vitro (Figure 6a–c, Supplementary Figure S2a–c). A dose-dependent decrease in RAB38 protein levels was observed in all cell lines (Figure 6d,g,j, Supplementary Figure S2d,f,h). Treatment of glioblastoma cells with simvastatin or lovastatin also produced a consistent decrease in c-Myc, Mcl-1, Bcl-2, and Bcl-xL levels (Figure 6e,f,h,i,k,l, Supplementary Figure S2e,g,i). Annexin V/PI assays were performed using LN229 cells that overexpress Bcl-xL, in order to determine if Bcl-xL has a role in the cell death induced by simvastatin (Figure 6m,n). The LN229 control group showed approximately 94% viable cells with approximately 2% of cells in early apoptosis and 3% of cells in late stages of cell death at 72 h (Figure 6m). In contrast, LN229 cells exposed to simvastatin at the concentration of 5 µM exhibited approximately 51% viability with 18% and 29% of cells in early and late stages of cell death, respectively (Figure 6m). The overexpression of Bcl-xL protected from cell death after simvastatin treatment (Figure 6m,n), with a peak of 82% healthy cells (Figure 6m).

In summary, we have shown that RAB38 is implicated in mediating glioblastoma growth and resistance to cell death and that this occurs by modulation of distinct cellular pathways.

## 4. Discussion

The RAB family of small GTPases is gaining increasing recognition for its critical role in regulating cancer progression [17,18,19]. The goal of this study was to investigate if RAB38 affects glioblastoma cell survival and proliferation and can present as a novel therapeutic target for glioblastoma. It has been established that RAB38 is upregulated in glioblastoma and associated with a poor prognosis and enhanced cell migration [9]. When comparing low-grade gliomas to glioblastomas, the latter demonstrated significantly higher RAB38 expression, which led to our use of the glioblastoma cell lines LN229, T98G, and the patient-derived GBM43 line. Despite evidence that RAB38 is upregulated in gliomas and correlated with a poor prognosis, the functional significance of RAB38 in glioblastoma growth and proliferation has not been studied prior to this investigation. RAB38 is a GTPase protein noted to be associated with vesicle transport regulation and other cell-type-specific functions. RAB38 has been previously noted to be an important factor in melanogenesis [20] and a driver of malignant progression of pancreatic and bladder cancer [18,21]. However, the mechanisms are not understood.

This study suggests RAB38 is an important regulator of glioblastoma cell viability. All glioblastoma cell lines in this study showed RAB38 protein expression, albeit at different levels. The results confirm that the RAB38 protein is expressed in glioblastoma cells, reinforcing the notion that RAB38 is a viable, potential therapeutic target for glioblastoma. Our data demonstrate significant decreases in cell viability 72 h following RAB38 knockdown (Figure 1b–d) and 14 days after silencing (Figure 1e,f). Interestingly, non-neoplastic astrocytes did not show any sign of apoptosis after RAB38 knockdown (Figure 2a), demonstrating that RAB38 could be a selective therapeutic target for glioblastoma. Furthermore, in glioblastoma cell lines, a significant reduction in cell cycle progression induced by RAB38 knockdown (Supplementary Figure S1e,f) and an increase in cell death (Figure 2b–d) were demonstrated.

To gain insight into the pathways affected by RAB38 activity in the context of glioblastoma, we focused our attention on c-Myc, a transcriptional factor that plays an important role in the pathogenesis of many tumors, including glioblastoma [14,15]. Our GSEA revealed that MYC targets were amongst the most significant downregulated gene sets (Supplementary Figure S1a). Consistently, we showed that overexpression of c-Myc rescued from the reduction in proliferation after RAB38 silencing (Supplementary Figure S1h–j). Taken together, these results suggest that RAB38 regulates the cell cycle of glioblastoma cells in part through c-Myc.

Furthermore, our data support the conclusion that RAB38 is mechanistically involved in the regulation of mitochondrial-mediated cell death. The TMRE analysis demonstrates increased mitochondrial stress (Figure 2e–g). Significant decreases in the oxygen consumption rate and glycolytic activity (Figure 3b–i) suggest that RAB38 may regulate mitochondrial-mediated cell death in glioblastoma. Our findings also suggest that RAB38 interferes with mitochondrial respiration, with an impairment of oxidative metabolism and downregulation of genes related to mitochondrial metabolism (Figure 3). Previous studies have convincingly linked impaired mitochondrial metabolism with susceptibility to BH3-mimetics [16,22]. Specifically, one group has recently demonstrated that the IDH1 R132H mutation sensitizes leukemia cells to the cytotoxic effect of the selective Bcl-2 inhibitor ABT199. Mechanistically, this was linked to 2-HG, which accumulates in IDH1 mutated tumors. 2-HG suppresses mitochondrial metabolism at the level of complex IV. Inhibition of complex IV suffices to enhance the apoptotic effects of ABT199 [23]. Similarly, work from our own group demonstrated comparable findings in the context of Bcl-xL inhibition [11]. Another recent report linked heme biosynthesis and sensitivity of leukemia cells to ABT199 [24]. In agreement with the findings above, inhibition of mitochondrial respiration/ATP synthesis is sufficient to enhance BH3-mimetic mediated cell death or loss in cellular viability (Figure 5). Our findings with RAB38 and its impact on mitochondrial metabolism and subsequent enhancement of ABT263-mediated cell killing are thus very much in line with these prior studies, but our findings related to the role of RAB38 in modulating these processes have not been described before (Figure 2, Figure 3 and Figure 5).

To provide more insights into the potentially involved pathways activated or inhibited through RAB38 inhibition, we have included our entire gene set enrichment analysis (Supplementary Table S1). As expected, several pathways were modulated after RAB38 silencing. Apoptosis, metabolism, and c-Myc pathways had an FDR q-value of less than 0.05, indicating statistical significance, which we also considered as relevant for glioma biology. We have confirmed through functional analysis that our selected pathways are either activated (apoptosis) or inhibited (MYC signaling and metabolism) following RAB38 silencing. It is well known that Bcl-xL is a bona fide inhibitor of mitochondrial-mediated intrinsic apoptotic cell death [25]. RAB38 is synthetically lethal with Bcl-xL inhibition through ABT263 (Figure 5). Moreover, Bcl-xL protein levels show a consistent decrease after RAB38 disruption in all cell lines (Figure 4a–c). After RAB38 knockdown, cells overexpressing Bcl-xL show rescued viability and reduced cell death (Figure 4e–h). Taken together, our data suggest that Bcl-xL is likely involved in the cell death induced by RAB38 silencing and protects glioblastoma cell lines from cell death.

All RABs require post-translational prenylation for membrane anchoring and function [26,27,28,29,30]. In the nonprenylated state, RABs are predominantly cytosolic and susceptible to rapid degradation. Newly synthesized RABs bind to an escort protein (RAB escort protein (REP); REP1 or REP2) and are then prenylated by the enzyme RAB geranylgeranyl transferase (RGGT) on their C-terminal Cys residue(s), which permits their specific membrane localization [28,30]. Interestingly, individual RABs exhibit wide variability in their extent of in vivo prenylation [30]. RAB38 is one of the slowest RABs to be prenylated both in vitro and in vivo, and its subcellular localization and function are rapidly altered by prenylation inhibitors and/or genetic disruption of REP or RGGT [28,31,32]. RAB38 mutation in mice and rats produces an in vivo phenotype that is very similar to that of RGGT and REP mutations in animals and humans [10,33]. In total, these observations suggest that RAB38 may be particularly sensitive to prenylation inhibition and, thus, be a target for in vivo glioblastoma therapy. RAB38-specific pharmacological inhibitors are under active development but are not yet in human use. However, RAB38 function can be inhibited using currently available pharmacological agents. Statins, which are FDA-approved and in wide clinical use, are known to inhibit RAB prenylation [34] and have been shown in a few previous studies to enhance temozolomide-induced glioblastoma cell death [35,36,37] and to induce a significant reduction in tumor volume in vivo [38,39,40,41,42]. Our studies demonstrated that treatment of glioblastoma cells with simvastatin or lovastatin causes a dose-dependent decrease in RAB38 expression and cell viability in LN229, T98G, and GBM43 cells in vitro (Figure 6, Supplementary Figure S2), which to the best of our knowledge has not been reported previously. Moreover, statins decreased expression of antiapoptotic Bcl-2 family members (Mcl-1, Bcl-2, and Bcl-xL) and c-Myc, in keeping with the effects elicited by specific silencing of RAB38, suggesting that interference with RAB38 partially phenocopies the molecular effects of statins. These results support the notion that statins might elicit their effects at least in part through modulation of RAB38. Although the mechanisms by which statins cause glioblastoma cell death are likely multiple, our hypothesis is that statin inhibition of RAB38 prenylation may be one element mediating their glioblastoma cytotoxic action. However, since statins are FDA-approved and widely used in humans, clinical studies with statins in glioblastoma patients could proceed rapidly. From our currently available data, it appears most likely that RAB38 silencing is sufficient to induce cell death in glioblastoma cell lines but RAB38 suppression is not necessary for the killing effects of statins. In conclusion, our study demonstrates that RAB38 is a critical regulator of cell survival in glioblastoma in vitro and strongly indicates RAB38 as a potential therapeutic target singly or in combination therapies for glioblastoma treatment.

## Data Availability

All the data are available upon request.

## Supplementary Materials

The following are available online at https://www.mdpi.com/article/10.3390/cells10071643/s1, Figure S1: RAB38 silencing regulates c-Myc expression and loss of RAB38 mediates inhibition of proliferation of glioblastoma cells in part through c-Myc, Figure S2: Lovastatin causes reduction in cellular viability and downregulates RAB38, RAB32, Mcl-1, Bcl-2, Bcl-xL, and c-Myc protein expression in human glioblastoma cells.
