# Supplementary material for: RAB38 Facilitates Energy Metabolism and Counteracts Cell Death in Glioblastoma Cells
Source: Cells. 2021 Jun 30;10(7):1643. doi: 10.3390/cells10071643 (PMC8306361; doi:10.3390/cells10071643)
Supplement: Supplementary file 1 [file cells-10-01643-s001.zip › cells-1233295 supplementary final/cells-1233295 supplementary figures final.pdf]

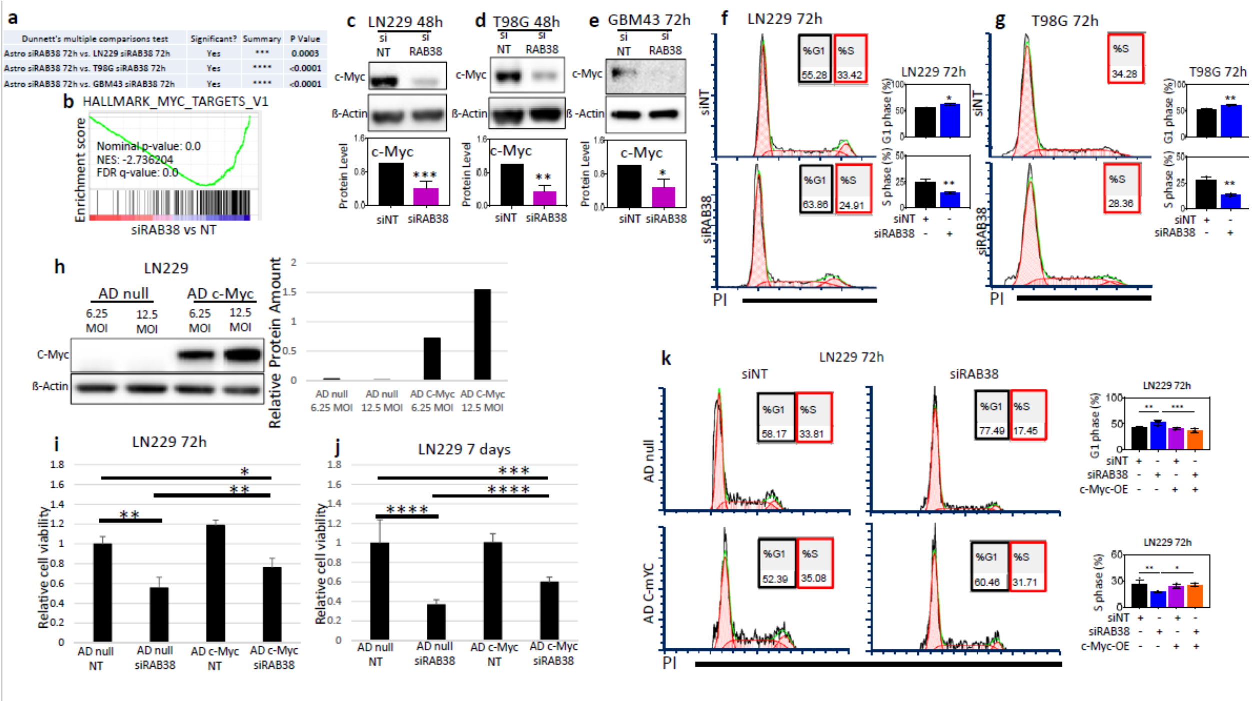

Suppl. Fig. 1. RAB38 silencing induces a significant difference in cellular viability in astrocytes versus the three GBM lines tested, regulates c-Myc expression and loss of RAB38 mediates inhibition of proliferation of glioblastoma cells in part through c-Myc. (a). Anova coupled with a multiple comparison between all the cell lines tested in Figure 1. **(b).** LN229 cells were transfected with non-targeting or RAB38 siRNA. RNA was isolated 48h after transfection and samples were submitted for transcriptome analysis and subsequently analyzed by GSEA. Shown is a GSEA plot with the respective statistical analysis suggesting a downregulation of c-Myc targets after RAB38 impairment. Data are presented as mean and SD, n=2. **(c-e).** RAB38 and respective control siRNA knockdown was performed in LN229 (c) and T98G (d) and GBM43 (e). Whole cell protein extracts were examined by western blot analysis of RAB38 and c-Myc. ß-actin western blot analysis was performed to confirm equal protein loading. **(f-g).** 72h following transfection samples were stained with PI and cell cycle analysis was performed. Representative plots and histograms with statistical analysis are presented (n=3). **(h).** Adenovrial mediated c-Myc overexpression was performed in LN229 and whole cell protein extracts were examined by western blot analysis of c-Myc and ß-actin. Bar graphs display protein quantification levels determined by ImageJ. **(i-j).** RAB38 was silenced in LN229 with non-targeting (NT) RNA or siRAB38 using cells over-expressing c-Myc versus control, for 72h (i) and for 7 days (j). Cellular viability was determined by CellTiter-Glo assay and relative cell viability was calculated. Data are presented as mean

and SD, n=3. **(k).** LN229 glioblastoma cell line over-expressing c-Myc or an appropriate related control were transfected with either NT or RAB38 siRNA and stained with propidium iodide at 72h and analyzed by flow cytometry. Representative plots and a histogram with statistical analysis are presented (n=3). *P < 0.05; **P < 0.01; ***/****P < 0.001.

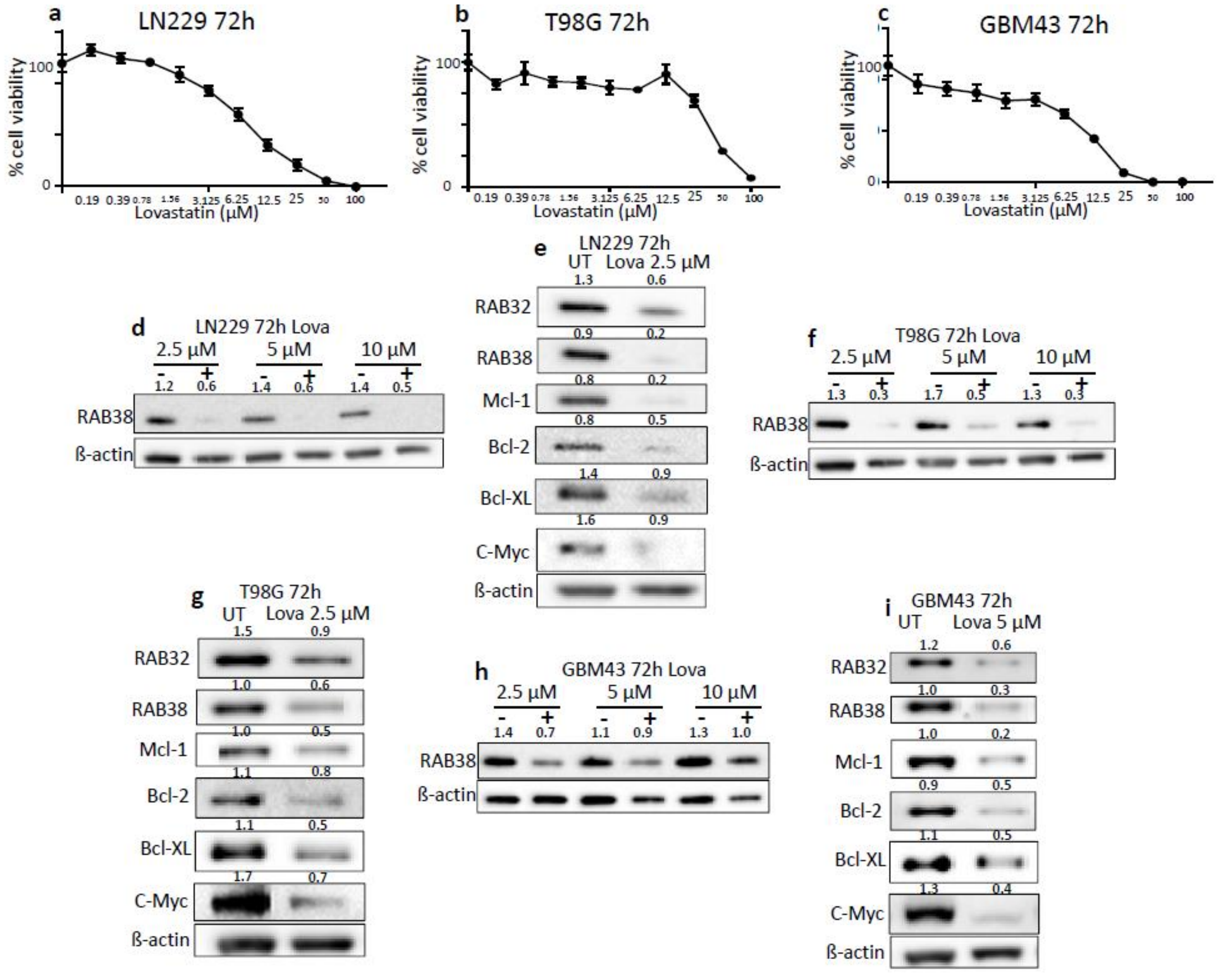

Suppl. Fig. 2. Lovastatin causes reduction in cellular viability and downregulates RAB38, RAB32, Mcl-1, Bcl-2, Bcl-xL and c-Myc protein expression in human glioblastoma cells. LN229 (a), T98G (b) and GBM43 (c) glioblastoma cell lines were treated with increasing concentrations of Lovastatin for 72h. Cellular viability was determined by CellTiter-Glo assay and the IC50-values were calculated based on a non-linear regression analysis. Data are presented as mean and SD, n=3. **(d-i).** LN229 (d,e), T98G (f,g) and GBM43 (h,i) were treated with Lovastatin at the indicated concentrations for 72h. Whole-cell protein extracts were examined by western blot for RAB38, RAB32, Mcl-1, Bcl-2, Bcl-xL and c-Myc. ß-actin western blot analysis was performed to confirm equal protein loading. n=3.

Suppl. Table 1. Gene set enrichment analysis of siRAB38 in LN229 cells
